# Parents' Perceptions and Intention to Vaccinate Their Children Against COVID-19: Results From a Cross-Sectional National Survey in India

**DOI:** 10.3389/fmed.2022.806702

**Published:** 2022-05-18

**Authors:** Bijaya Kumar Padhi, Prakasini Satapathy, Vineeth Rajagopal, Neeti Rustagi, Jatina Vij, Lovely Jain, Venkatesan Chakrapani, Binod Kumar Patro, Sitanshu Sekhar Kar, Ritesh Singh, Star Pala, Lalit Sankhe, Bhavesh Modi, Surya Bali, Tanvi Kiran, Kapil Goel, Arun Kumar Aggarwal, Madhu Gupta

**Affiliations:** ^1^Department of Community Medicine and School of Public Health, Postgraduate Institute of Medical Education and Research (PGIMER), Chandigarh, India; ^2^Department of Public Health, Utkal University, Bhubaneswar, India; ^3^Department of Community Medicine & Family Medicine, All India Institute of Medical Sciences (AIIMS), Jodhpur, India; ^4^Centre for Sexuality and Health Research and Policy (C-SHaRP), Chennai, India; ^5^Department of Community and Family Medicine, All India Institute of Medical Sciences (AIIMS), Bhubaneswar, India; ^6^Department of Preventive and Social Medicine, Jawaharlal Institute of Postgraduate Medical Education & Research (JIPMER), Puducherry, India; ^7^Department of Community and Family Medicine, All India Institute of Medical Sciences (AIIMS), Kalyani, India; ^8^Department of Community Medicine, North Eastern Indira Gandhi Regional Institute of Health & Medical Sciences (NEIGRIHMS), Shillong, India; ^9^Grant Medical College & JJ Hospital, Mumbai, India; ^10^Department of Community and Family Medicine, All India Institute of Medical Sciences (AIIMS), Rajkot, India; ^11^Department of Community and Family Medicine, All India Institute of Medical Sciences (AIIMS), Bhopal, India

**Keywords:** vaccine acceptance, vaccine hesitancy, children, risk perception, trust

## Abstract

**Background:**

Despite the success of adult vaccination against COVID-19, providing vaccines to children remains a challenge for policymakers globally. As parents are primary decision-makers for their children, we aimed to assess parents' perceptions and intentions regarding COVID-19 vaccination in India.

**Methods:**

A cross-sectional web-based study was designed, parents or caregivers (*N* = 770) were recruited through snowball sampling using Google form. Cross-tabulation was performed by parents' intention to vaccinate their children against COVID-19 virus with sociodemographic characteristics and their risk perception toward COVID-19, trust in the healthcare system, and their history of vaccine hesitancy behavior. Multivariable logistic regression analysis was performed to compute the predictors of child vaccination intention among Indian parents.

**Results:**

Seven hundred and seventy parents across the country have completed the survey. Of the 770 participants, 258 (33.5%) have shown intent to vaccinate their children. The stated likelihood of child vaccination was greater among parents who had a bachelor's degree or higher education (aOR: 1.98, 95% CI: 1.15–3.51); as well as among parents who intended to vaccinate themselves (aOR: 2.35, 95% CI: 1.30–4.67). Parental concerns centered around vaccine safety and side effects.

**Conclusion:**

Indian parents reported high knowledge of the COVID-19 virus and were aware of the development of a vaccine. However, about one-third of parents intended to vaccinate their children, and about half of them were not sure whether to vaccinate their children or not against the COVID-19 virus. The study highlighted the need for health promotion strategies that promote vaccine uptake among parents.

## Introduction

The World Health Organization (WHO) has reported more than 242 million confirmed cases of COVID-19 worldwide, including ~5 million deaths as of 20 October 2021 (1). India reported 34 million confirmed cases and 450 thousand deaths as of 25th October 2021 (1). An effective strategy to mitigate the morbidity and mortality of the COVID-19 and to ensure population higher levels of immunity is the development of an effective and safe vaccine for all populations, including children. In general, children with COVID-19 present with milder symptoms and are at lower risk of hospitalization and life-threatening complications (2). Numerous public health authorities, including the WHO and United States Centers for Disease Control and Prevention, advocate a vaccine for children. However, acceptance of the COVID-19 vaccine among parents or caregivers remains unexplored.

Since the start of the COVID-19 pandemic, a high number of vaccines is being developed (3). The WHO has declared on the 11th of July 2021 that 107 and 184 vaccine candidates are in clinical and pre-clinical stages, respectively, and at least 13 vaccines have been approved and administrated through four platforms (4). As of 25 October 2021, ~6 billion vaccine doses have been administrated worldwide (1). In India, as of 25 October 2021, the Ministry of Health and Family Welfare reported over one billion doses to adults (5). To expand the vaccine administration to children, the government of India is planning to implement a COVID-19 vaccine campaign for the age group of 12–18 years old (6).

Numerous studies on the COVID-19 vaccine acceptance shows that a lower number of parents were interested in vaccinating their children (7–15). A national survey in the United States reported less than one-half of parents are likely to administer a COVID-19 vaccine to their children (16). In Canada, 63.0% of parents intend to vaccinate their children against COVID-19 (10). In Turkey, only 36.3% of parents were willing to have their children receive the COVID-19 vaccine (13). In China, COVID-19 vaccine hesitancy was found to be 8.4% among reproductive women (8). In Japan, 472 (42.9%) out of 1,100 respondents, were willing to vaccinate their children (15). In a hospital-based study in Saudi Arabia, one-fourth of mothers were hesitant toward childhood immunization (11). To the best of our knowledge, none of the studies conducted in India address the intention and perception of parents' willingness to vaccinate their children in India.

Despite the success of adult vaccination globally, childhood immunization against the COVID-19 vaccine is now a challenge for policymakers. Including an effective and safe vaccine, a high level of vaccine acceptance among the parents or caregivers is also a key factor for global immunization success. Many factors such as fear about the vaccine safety and risk for a child to be infected by the virus impact parents' acceptance of the new COVID-19 vaccination program (13). Therefore, understanding parents' perception and acceptance of the COVID-19 vaccine will help in designing effective strategies for promotion and rolling out COVID-19 vaccination and beyond. To the best of our knowledge, this is the first community-based study in India that investigates parental willingness for COVID-19 vaccination. The study also explored factors associated with vaccine hesitancy among Indian parents or caregivers.

## Methods

### Study Design and Sample

We conducted a national online cross-sectional survey between November 2020 and January 2021 (before the introduction of the COVID-19 vaccine). The overall sample (*N* = 770) of parents were recruited across major geographical regions in India with an equal weightage to the urban and rural population. Respondents were adults who have one or more children 0–17 years old in their home and have access to the internet or telephone. We estimated a minimal sample size of 768, based on the maximum variability possible in the outcome variable in the population (i.e., a proportion of 0.50), with a margin of error of ±5 and 95% confidence intervals (CI). The design effect was kept at 2. Study participants were recruited through snowball sampling. Invitations to participate in the study were distributed to the respondents *via* email, social media: Twitter, Facebook, and the WhatsApp communication platform of the primary contacts of the study team and requested to transmit further for its maximum reach. Considerations were made to recruit the participants across major geographic regions of India. To ensure rigor and validity, respondents had unique IDs, and 10% of respondents were contacted by telephone for cross verification of the presence of children 0–17 years old in their home.

### Questionnaire Development

A first draft of the questionnaire was prepared based on a review of the relevant literature and reviewed by subject experts. The draft questionnaires were pilot tested with 50 participants and adjusted for accuracy and clarity. The consistency and stability of the final questionnaires were tested using Cronbach's alpha (0.7). The online survey was administered *via* Google Forms and was distributed to the parents *via* Facebook, WhatsApp, and mail groups. Informed consent was obtained from all parents (18 years or older and currently living in India). Detailed responses were recorded further only those who provided informed consent to complete the survey.

#### Sociodemographic Measures

The survey has detailed information about the sociodemographic characteristics of study participants, including parental age, sex, education level, place of residence, income, occupation, and social status in the community.

#### Parents Knowledge of COVID-19 and Vaccine Intention

The survey tested parents' knowledge of the COVID-19 virus by asking, “Before this interview, were you aware that the COVID-19 virus is currently circulating in the community?”. The responses were captured in a 3-point Likert scale (Yes, No, and Do not know). We also asked parents to record their response to vaccine development knowledge by asking, “To the best of your knowledge, is there currently a vaccine being prepared for the pandemic Coronavirus strain referred to as COVID-19 vaccine?”. Parents' own likelihood of getting a COVID-19 vaccine (same 3-point Likert scale: Yes, No, and Not sure) was also recorded.

#### Intent to Vaccinate the Child

The survey asked, “Do you intend to vaccinate your child(ren) for COVID-19 once a vaccine is available for children?”. The responses were captured in a 3-point Likert scale (Yes, No, and Not sure). We coded “Yes” as “likely to get a COVID-19 vaccine;” all others were labeled as “hesitant”.

### Statistical Analyses

The key outcome measures of the study were to know the parent's intention to vaccinate their children against the COVID-19 virus. Those who responded “Yes” were labeled as “Intended to vaccinate” vs. “hesitancy.” Descriptive statistical analysis was performed by doing cross-tabulation of demographic characteristics with the primary response variable “Intended to vaccinate.” We performed both simple and multivariable logistic regression analyses to compute the odds ratio (OR) and a 95% confidence interval (CI). Chi-squared tests were performed for bivariate analysis (cross-tabulation) between the outcome variable and all explanatory variables. A multivariate logistic regression model was constructed by including factors with *P* < 0.05 at binary comparisons. Inference on significant association was considered with a two-tailed *p* ≤ 0.05. STATA 15.0 software (StataCorp LP, Texas, USA) was used for all statistical analyses.

### Ethical Considerations

Ethical approval was granted for the study by the institutional Research Ethics Committee of Post Graduate Institute of Medical Education and Research (PGIMER), Chandigarh, India. Informed assent was taken before participation in the study. Anonymized data was used for analysis, interpretation, and reporting.

## Results

Seven hundred and seventy parents participated in this study. Over half of the parents were 30–49 years old (60.0%), 39.6% were female, 28.5% were educated to graduate level or higher, 23.4% employed in government sector, 59.7% had more than six members in their family, 31.8% had a monthly income above 50,000 Indian rupees, 41.3% reside in rural areas, and 48.2% perceived their social status as high in the community (Table 1).

**Table 1:** Sociodemographic characteristics of the study respondents (*N* = 770).

**Variables**	***n* (%)**
**Parental age**	
18–29	120 (15.6)
30–49	462 (60.0)
Above 50	188 (24.4)
**Parental sex**	
Male	465 (60.4)
Female	305 (39.6)
**Parental education**	
No formal education	26 (3.4)
Primary school	193 (25.1)
Diploma/high school	262 (34.0)
Undergraduate	130 (16.9)
Postgraduate and above	89 (11.6)
**Parent's employment status**	
Government sector	180 (23.4)
Private sector	282 (36.6)
Self-employed	230 (29.9)
Unemployed	78 (10.1)
**Family size**	
Five and below	310 (40.3)
Six and above	460 (59.7)
**Family income (monthly, INR)**	
Below 10,000	52 (6.8)
11,000–20,000	291 (37.8)
21,000–50,000	182 (23.6)
Above 5,0000	245 (31.8)
**Social status**	
Low	108 (14.0)
Medium	291 (37.8)
High	371 (48.2)
**Region of residence**	
Eastern	192 (24.9)
Western	91 (11.8)
Northern	210 (27.3)
Southern	108 (14.0)
Central	86 (11.2)
North-east	83 (10.8)
**Area of residence**	
Urban	452 (58.7)
Rural	318 (41.3)
**Social caste**	
Other backward caste	312 (40.5)
Unreserved	215 (27.9)
Scheduled caste	150 (19.5)
Scheduled tribe	93 (12.1)
**Religion**	
Hindu	367 (47.7)
Muslim	173 (22.5)
Christian	85 (11.0)
Sikhs	105 (13.6)
Other	40 (5.2)

The prevalence of parents' willingness to allow their children to receive the COVID-19 vaccine was 33.5%, whereas 40.3% of parents were willing to receive the COVID-19 vaccine. At the time of survey, 27.5% of parents had a history of exposure to confirmed COVID-19 cases and 62.7% had concerned of getting infected by COVID-19 virus. Most (88.3%) of the parents were aware that COVID-19 virus is currently circulating in the community, and 86.0% of them had knew that a vaccine is being prepared for the pandemic Coronavirus strain referred to as “COVID-19 vaccine.” Of the 770 parents, about one-fourth of them had a previous history of vaccine hesitancy, 62.2% had trusted the healthcare system, and 39.1% considered domestic vaccines were good (Table 2).

**Table 2:** Willingness to accept COVID-19 vaccine, contact history with COVID-19 patients, risk perception, and vaccine preferences among parents (*N* = 770).

**Variables**	***n* (%)**
Intended to vaccinate the child	
Yes	258 (33.5)
No	164 (21.3)
Not sure	348 (45.2)
Intended to vaccinate themselves	
Yes	310 (40.3)
No	270 (35.1)
Not sure	190 (24.7)
Exposed to COVID-19 cases	
No	212 (27.5)
Yes	558 (72.5)
Knowledge about the COVID-19 virus	
No	90 (11.7)
Yes	680 (88.3)
Knowledge about the development of the COVID-19 vaccine	
No	108 (14.0)
Yes	662 (86.0)
History of vaccine hesitancy	
Yes	210 (27.3)
No	560 (72.7)
High-risk perception	
Yes	483 (62.7)
No	287 (37.3)
Trust in the healthcare system	
No	291 (37.8)
Yes	479 (62.2)
Trust in domestic vaccines	
Better	301 (39.1)
Neutral	282 (36.6)
Worse/not sure	187 (24.3)

Table 3 shows the analysis of bivariate and multivariable logistic models, and determined the predictors affecting parents' willingness to allow their children to be given the COVID-19 vaccine. In the bivariate analyses, trust in the healthcare system and domestic vaccines, higher risk perception, being employed in the government sector, and being a rural resident or a female were found to be associated with parent's intention to vaccinate their children. After adjusting for confounding variables, parent's own willingness to receive the COVID-19 vaccine (aOR: 2.35, 95% CI: 1.30–4.67), and parents who had a bachelor's degree or higher education (aOR: 1.98, 95% CI: 1.15–3.51) were found to be associated with parents' willingness to vaccinate their children (Table 3).

**Table 3:** Unadjusted and adjusted odds ratios for the association between parent's intention to vaccinate their children with sociodemographic and other COVID-19 behaviors (*N* = 770).

	**“Intended to vaccinate children”**		
**Variable**	**OR [95% CI]**	**aOR [95% CI]**	***P*-value**
**Parents' COVID-19 vaccination intention for themselves**			
No	Ref	Ref	
Yes	2.88 [1.21–4.06]	2.35 [1.30–4.67]	0.001
**Exposed to COVID-19 cases**			
No	Ref	Ref	
Yes	1.71[0.81–2.61]	1.28 [0.46–2.25]	0.311
**Trust in the healthcare system**			
No	Ref	Ref	
Yes	2.42 [1.41–3.55]	2.11 [0.98–3.17]	0.068
**History of vaccine hesitancy**			
Yes	Ref	Ref	
No	1.48 [0.81–3.66]	1.45 [0.27–2.74]	0.241
**Higher risk perception**			
No	Ref	Ref	
Yes	2.46 [1.19–3.80]	1.87 [0.99–3.17]	0.067
**Opinion on domestic vaccines**			
Worse	Ref	Ref	
Better	2.72 [1.02–5.08]	1.67 [0.71–3.97]	0.165
Neutral	2.30 [1.04–2.87]	1.40 [0.26–2.83]	0.151
**Age**			
18–29	Ref	Ref	
30–49	1.93 [0.85–2.23]	1.13 [0.48–2.77]	0.126
Above 50	1.78 [0.91–2.14]	1.52 [0.35–2.17]	0.132
**Sex**			
Male	Ref	Ref	
Female	1.97 [1.07–2.20]	1.35 [0.97–2.44]	0.081
**Employment status**			
Unemployed/self-employed	Ref	Ref	
Private sector	1.51 [0.65–2.20]	1.18 [0.31–2.70]	0.142
Government sector	1.91 [1.07–3.31]	1.30 [0.71–2.67]	0.095
**Education**			
Primary or below	Ref	Ref	
Diploma/High School	2.18 [1.19–5.82]	1.86 [0.95–4.61]	0.087
Undergraduate and higher	3.41[1.32–6.11]	1.98 [1.15–3.51]	0.001
**Social status in the community**			
Low	Ref	Ref	
Medium	1.80 [0.59–2.72]	1.57 [0.92–2.67]	0.085
High	1.83 [0.58–2.88]	1.16 [0.75–2.24]	0.116
**Place of residence**			
Urban	Ref	Ref	
Rural	1.92 [1.07–3.12]	1.25 [0.44–3.05]	0.134

**Figure 1:**
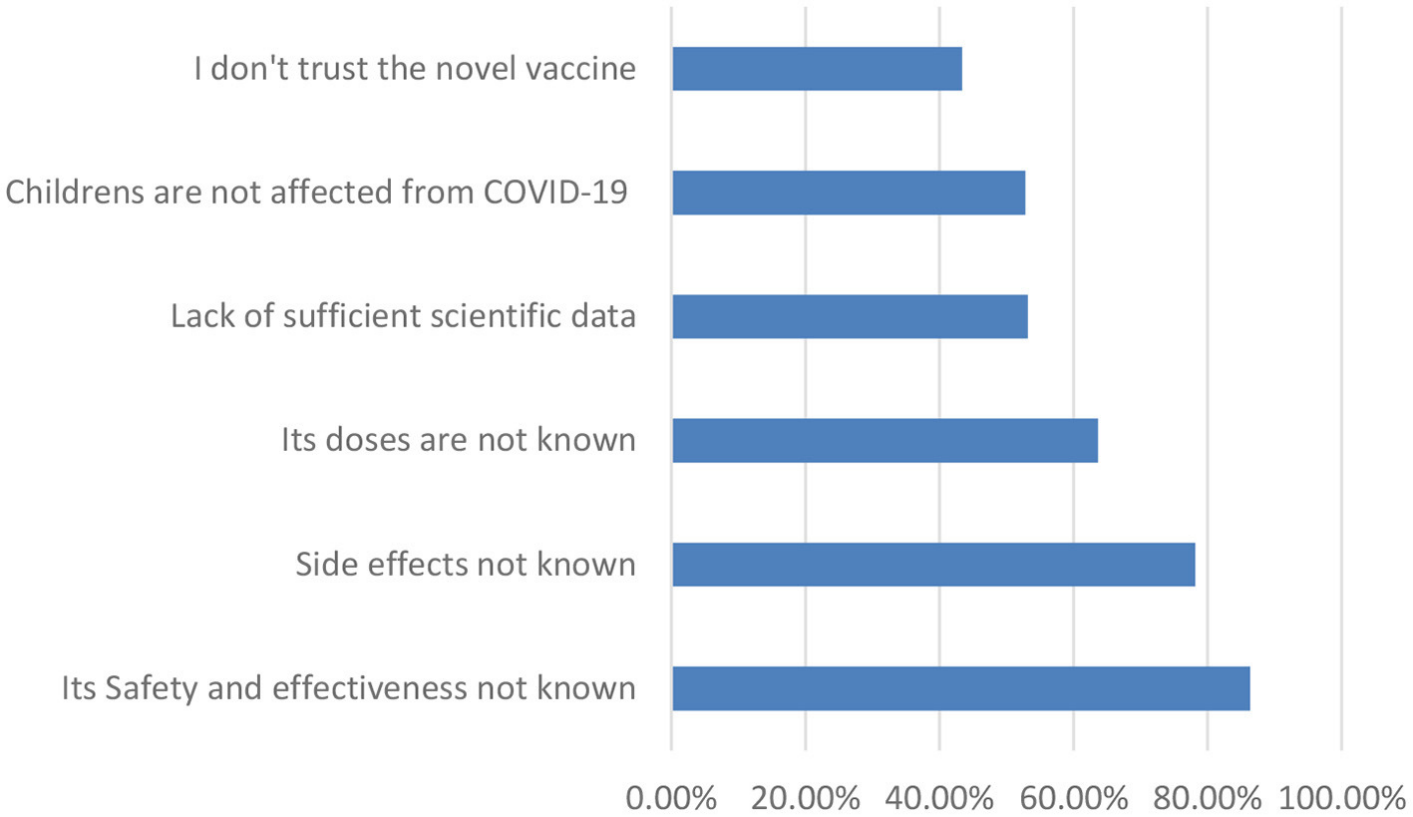
Parents' concerns for not willing to vaccinate of their children for COVID-19 (*n* = 770).

Figure 1, shows the concerns of parents in accepting the novel COVID-19 vaccine. The key reasons for reluctance to vaccinate their children were: concerns about safety and effectiveness (86.4%), fear of side effects (78.2%), lack of information about doses (63.7%), lack of sufficient scientific data (53.2%), perception that children are not affected by COVID-19 (52.8%), and lack of trust in vaccine (43.4%).

## Discussion

In this study, we aimed to determine the acceptability of the COVID-19 vaccine among parents in India and the associated factors. To the best of our knowledge, this is the first study in India that investigates parents' willingness to administer COVID-19 vaccine to their children. Understanding the factors associated with vaccination intentions will help in implementation of vaccination program and elsewhere. We found that nearly 33.5% of parents would be willing to administer a COVID-19 vaccine to their child. Sociodemographic factors, such as higher education level and parents who intended to vaccinate themselves, were found to be associated with childhood vaccination. The main concerns raised by parents in our study were around the safety and effectiveness of the COVID-19 vaccine.

Our findings contribute to an emerging literature suggesting that there is a low demand for childhood COVID-19 vaccine among parents (7–9, 11, 17). A study conducted in Texas, USA reported that about 38.3% of mothers had no intention to consider COVID-19 vaccine for their children (17). The proportion of willing parents in India is considerably lower as compared to other countries as per studies from England (89%) (7); New Zealand (80%) (18); China (73%) (19); USA (65%) (20); Canada (63%) (10); Japan (42.9%) (15) and Turkey (42%) (21). A large proportion of participants in our study were aware about COVID-19 virus (88.3%); developmental status of COVID-19 vaccine (86%) and were exposed to COVID-19 (72.5%). In spite of that, less than half of the participants were willing to get themselves readily vaccinated (40.3%). This supports the findings that parent's intention to themselves get vaccinated is the most influential factor determining their intent toward children vaccination. Factors such as newness of vaccine, rapid development and unknown long-term side effects influence parent's perception of vaccine safety and their intention to get themselves and their children vaccinated (13). As reported by numerous studies, vaccine safety is fundamental to maintaining the public's trust in vaccines (12, 22, 23). Thus, future studies are required to understand long-term change in the perception of parents and factors influencing their intention to get themselves and their children vaccinated. Health care providers play a key role in influencing decision of parents toward vaccine acceptance and uptake. It is essential to thus engage with various level of health care providers and strategize vaccine messages to parents, especially those from lower education status. In our study the variable with the highest aOR [2.35; 95% CI: 1.30–4.67] was “parents' vaccination intention for themselves.” One possible interpretation of this result could be that if parents will (not) vaccinate themselves they will (not) vaccinate their children. So the hesitancy regarding vaccinating their children may not be due to special concerns about effectiveness and safety of the vaccine for children as compared to adults, but rather an overarching disbelief in the safety and effectiveness of the vaccine for anyone, adults or children. Therefore, emphasis should be drawn on the safety and effectiveness of vaccines in the rollout program.

Limitations of this study that should be considered include the sampling strategy adopted by the study which may not be representative of all parents in the country, limiting the generalizability of our finding. The cross-sectional design of the study should be interpretated carefully when accessing overall prevalence of vaccine hesitancy among parents in India. Our sample represented individuals with internet access and having mobile literacy. This does not necessarily reflect the country-wide perception of parents toward vaccine and their intention to get children vaccinated.

Despite the above limitations, our study assessed nationally representative sample of parents regarding their perceptions and intentions to vaccinate their children at a critical time, just as adult COVID-19 vaccination programs were initiated in India. Our findings reminiscent of recent research suggesting that there is a low demand for COVID-19 vaccine among parents, and highlights the need for longitudinal studies to measure the acceptability of a COVID-19 vaccines at different intervals. Future studies are thus required to supplement our current findings to enhance vaccine uptake among children in India. Nevertheless, our study establishes evidence regarding parent's hesitancy toward getting their children vaccinated and their concerns in the coming months must be addressed through targeted communication strategies.

## Conclusion

This study depicts that Indian parents have good knowledge of COVID-19 and vaccine developments. However, parents' intention to vaccinate their children was found to be low, due to concerns about vaccine safety. The study warrants implementation of strategies to improve parents' knowledge about the safety, efficacy and side effects of COVID-19 vaccine.

## Data Availability Statement

The raw data supporting the conclusions of this article will be made available by the authors, without undue reservation.

## Ethics Statement

The studies involving human participants were reviewed and approved by Institutional Ethical Committee of Post Graduate Institute of Medical Education and Research (PGIMER), Chandigarh, India. The patients/participants provided their written informed consent to participate in this study.

## Author Contributions

BP, MG, and AA conceptualized the study and designed the tools. BP, LJ, JV, PS, VC, BP, SK, RS, SP, SB, NR, VR, TK, KG, BM, LS, MG, and AA conducted the study at national level and collected the data. All authors reviewed drafts, provided edits, and approved the final submission.

## Conflict of Interest

The authors declare that the research was conducted in the absence of any commercial or financial relationships that could be construed as a potential conflict of interest.

## Publisher's Note

All claims expressed in this article are solely those of the authors and do not necessarily represent those of their affiliated organizations, or those of the publisher, the editors and the reviewers. Any product that may be evaluated in this article, or claim that may be made by its manufacturer, is not guaranteed or endorsed by the publisher.

## Abbreviations

COVID-19: Coronavirus disease-2019
WHO: World Health organization.
